# Overexpression of SP1 restores autophagy to alleviate acute renal injury induced by ischemia-reperfusion through the miR-205/PTEN/Akt pathway

**DOI:** 10.1186/s12950-021-00270-y

**Published:** 2021-02-05

**Authors:** Chong Huang, Yan Chen, Bin Lai, Yan-Xia Chen, Cheng-Yun Xu, Yuan-Fei Liu

**Affiliations:** 1 Department of Nephrology, The Second Affiliated Hospital of Nanchang University, 330006 Nanchang, Jiangxi Province People’s Republic of China; 2 Department of Gastrointestinal Surgery, The Second Affiliated Hospital of Nanchang University, 330006 Nanchang, Jiangxi Province People’s Republic of China; 3 Department of Emergency, The Second Affiliated Hospital of Nanchang University, No.1, Minde Road, 330006 Nanchang, Jiangxi Province People’s Republic of China

**Keywords:** SP1, miR-205, PTEN, Autophagy, Ischemia/reperfusion injury

## Abstract

**Background:**

Acute kidney injury (AKI) is a major kidney disease with poor clinical outcome. SP1, a well-known transcription factor, plays a critical role in AKI and subsequent kidney repair through the regulation of various cell biologic processes. However, the underlying mechanism of SP1 in these pathological processes remain largely unknown.

**Methods:**

An *in vitro* HK-2 cells with anoxia-reoxygenation injury model (*In vitro* simulated ischemic injury disease) and an *in vivo* rat renal ischemia-reperfusion injury model were used in this study. The expression levels of SP1, miR-205 and PTEN were detected by RT-qPCR, and the protein expression levels of SP1, p62, PTEN, AKT, p-AKT, LC3II, LC3I and Beclin-1 were assayed by western blot. Cell proliferation was assessed by MTT assay, and the cell apoptosis was detected by flow cytometry. The secretions of IL-6 and TNF-α were detected by ELISA. The targeted relationship between miR-205 and PTEN was confirmed by dual luciferase report assay. The expression and positioning of LC-3 were observed by immunofluorescence staining. TUNEL staining was used to detect cell apoptosis and immunohistochemical analysis was used to evaluate the expression of SP1 in renal tissue after ischemia-reperfusion injury in rats.

**Results:**

The expression of PTEN was upregulated while SP1 and miR-205 were downregulated in renal ischemia-reperfusion injury. Overexpression of SP1 protected renal tubule cell against injury induced by ischemia-reperfusion via miR-205/PTEN/Akt pathway mediated autophagy. Overexpression of SP1 attenuated renal ischemia-reperfusion injury in rats.

**Conclusions:**

SP1 overexpression restored autophagy to alleviate acute renal injury induced by ischemia-reperfusion through the miR-205/PTEN/Akt pathway.

## Background

Acute kidney injury (AKI) is a clinical syndrome with acute renal insufficiency, which causes obvious water-electrolyte disorder, acid-base imbalance, and azotemia [1, 2]. AKI can be induced by many factors such as ischemia-reperfusion (I/R), drug toxicity and sepsis [3]. AKI often develops into chronic kidney disease [4]. Renal I/R injury is a frequent cause of AKI [5] and a major cause of renal transplant dysfunction [6]. The kidney is particularly prone to ischemia/hypoxic damage, and the oxygen flow of the kidney is also the highest organ in the body. The kidney needs the active reabsorption function of the renal tubules to complete its function of removing metabolites, and the maintenance of the physiological functions of the renal tubules requires a large amount of oxygen. Usually, the reabsorption of more than 99 % of the sodium in the renal tubules needs to consume the adenosine triphosphatase (ATP) produced by the cell mitochondria. There is an obvious imbalance between the blood supply and oxygen supply of the kidney [7]. Studies have shown that there is intrarenal/renal interstitial hypoxia in the kidneys. Hypoxia can lead to abnormal metabolism, biochemical disorders, structural and functional impairments of renal tubular epithelial cells, induce inflammatory reactions, and generate oxygen free radicals, which can cause, aggravate, or amplify the pathological changes of the kidney caused by chronic hypoxic injury [8, 9]. Therefore, ischemia and hypoxia play a very important role in the occurrence and development of kidney disease.

AKI induced by I/R has a complex pathogenesis involving innate and adaptive immune responses [10]. Studies have shown that AKI autophagy induced by I/R plays an important role in AKI *in* *vivo* and *in* *vitro* [11, 12]. Autophagy is induced under a variety of stress conditions such as cell starvation, growth factor deprivation, and enhanced autophagy has been shown to protect renal tubular epithelial cells by reducing I/R-induced apoptosis [13]. SP1 is a ubiquitous transcription factor that can participate in the regulation of cell proliferation, apoptosis, cell cycle and autophagy and other biological processes [14–16]. Studies have shown that increasing the expression of SP1 played a crucial role in protecting kidney I/R injury [17]. However, the pathogenic role of SP1 in AKI remains unclear.

MicroRNAs are epigenetic regulators of gene expression at the posttranscriptional level. MicroRNAs are involved in intercellular communication and crosstalk between different organs. As key regulators of homeostasis, dysregulation of microRNAs underlies several morbidities including kidney disease. In addition, their remarkable stability in plasma and urine makes them an attractive source of biomarkers. Increasing evidence suggests an interesting interaction between SP1 and microRNAs. In the study of prostate cancer, it was found that SP1 regulates the expression of miR-3178 and affects the metastasis of prostate cancer cells [18]. In esophageal squamous cell carcinoma, SP1 directly activates the expression of miR-205 to regulate the radiation sensitivity of cancer cells [19]. Furthermore, miR-205 activates Akt/autophagy pathway by targeting PTEN to promote angiogenesis of endothelial progenitor cells [20]. Moreover, miR-205 reduced hypoxia-induced renal cell injury through the PTEN/Akt signaling pathway [21], which may become a new potential target in the treatment of renal ischemia-reperfusion injury. Whether the overexpression of SP1 can activate autophagy through miR-205/PTEN/Akt signaling pathway to alleviate acute kidney injury caused by I/R has not been reported yet. Our study aims to explore whether the overexpression of SP1 can activate autophagy by mediating miR-205/PTEN/Akt signaling pathway to alleviate I/R induced AKI and provide novel therapeutic targets for prevention and therapy of I/R induced AKI.

## Methods

### Cell culture and I/R protocol

Human renal tubular epithelial cell (HK-2) was purchased from the American Type Cell Culture Collection (ATCC; Rockville, MD, USA). HK-2 cells were cultured in Dulbecco’s modified Eagle medium (DMEM, Gibco, Grand Island, NY, US) supplemented with 10 % fetal bovine serum (FBS, Gibco, Grand Island, NY, US) and 1 % penicillin-streptomycin solution (Gibco, Grand Island, NY, US) in an atmosphere containing 5 % CO_2_ at 37˚C and were sub-cultured every 3‑4 days after reaching 80 % confluence.

To simulate an anoxic environment, cells were cultured in serum-free DMEM in a three-gas incubator containing 94 % N_2_, 5 % CO_2_, and 1 % O_2_ for 24 h at 37˚C. Following exposure to hypoxic conditions, cell medium was replaced with fresh oxygenated DMEM and cells were reoxygenated (5 % CO_2_, 21 % O_2_, and 74 % N_2_) for 12 h at 37˚C.

### Cell transfection

The SP1 overexpression vector, miR-205 inhibitor, miR-205 mimic and negative control (NC) were synthesized by GenePharma (Shanghai, China). HK-2 cells were transfected with miR-205 inhibitor or mimic, SP1 overexpression vector or negative control using lipofectamine 2000 (Invitrogen, Carlsbad, CA, USA) according to the manufacturer’s instruction.

### Reverse‐Transcription Quantitative Polymerase Chain Reaction (RT-qPCR)

 Total RNA was extracted from cells with TRIzol reagent (Invitrogen, Carlsbad, CA, USA) according to the manufacturer’s instructions and stored at − 80˚C. Reverse transcription of RNA was done using Revert Aid™ First Strand cDNA Synthesis Kit (Invitrogen) according to manufacturer’s instruction. RT-qPCR was performed with the PrimeScript™ RT Master Mix kit (TaKaRa, China) on ABI system (Applied Biosystems, Life Technologies). Expression levels of genes were calculated with the 2^−△△Ct^ method using either U6 or GAPDH as internal control genes. The primer sequences used were: SP1 forward 5’-GACAGGACCCCCTTGAGCTT-3’ and reverse 5’-GGCACCACCACCATTACCAT-3’. PTEN forward 5’-CGACGGGAAGACAAGTTCAT-3’ and reverse 5’-AGGTTTCCTCTGGTCCTGGT-3’. miR-205 forward 5’-CGGTCCTTCATTCCACCGG-3’ and reverse 5’-GTCGTATCCAGTGCAGGGTCCGAGGTATTCGCACTGGATACGACCAGACT-3’. GAPDH forward 5’-CCAGGTGGTCTCCTCTGA-3’ and reverse 5’-GCTGTAGCCAAATCGTTGT-3’. U6 forward 5’-CTCGCTTCGGCAGCACA-3’ and reverse 5’-AACGCTTCACGAATTTGCGT-3’.

### Western blot

Cells in each group were lysed in the RIPA buffer (Sigma-Aldrich, Burlington, Massachusetts, USA), and the protein concentrations were determined using the BCA protein assay kit (ThermoFisher, Waltham, MA, USA). The proteins (20 µg) were resolved by 10 % SDS polyacrylamide gels and transferred onto a PVDF membrane which then were blocked with TBST buffer (20 mM Tris, 137 mM NaCl, 0.1 % Tween-20, pH 8.0) containing 5 % non-fat milk and incubated with the indicated primary antibody in Tris-buffered saline overnight at 4°C. The primary antibodies against SP1 (#5931), PTEN (#9559), AKT (#4691), p-AKT (#4060), LC3II/I (#4108), Beclin-1 (#3738), p62(#39,786) and GAPDH (#2118) were purchased from Cell Signaling Technology (CST, Danvers, MA, USA) and diluted following manufacturer’s instructions. Following extensive washing, the membranes were then incubated with the appropriate horseradish peroxidase-conjugated secondary antibody for 1 h at room temperature. Secondary antibodies used for western blot were goat-anti-rabbit (ProSci Inc., Poway, CA) and goat-anti-rat (Santa Cruz Biotechnology). The immunoreactivity was visualized by enhanced chemiluminescence (ThermoFisher, Waltham, MA, USA). The proteins were quantified using Quantity One software (Bio-Rad Laboratories, Inc., Hercules, CA, USA).

### 3-(4,5-Dimethylthiazol-2-yl)-2,5-diphenyltetrazolium bromide (MTT)

HK-2 cells were seeded into 96-well culture plates at a density of 3–5 × 10^3^ cells/well. After 24 h inoculation, 20 µL of MTT solution (5 mg/mL) was added into each well and cultured at 37°C for 2 h. The medium was discarded, and 150 mL dimethyl sulfoxide (DMSO) was added to each well to dissolve the formazan crystals. The mixture was shaken for 10 min to dissolve crystals, and the absorbance was measured at 490 nm using a microplate reader (Molecular Devices, USA) with the optical density (OD), and the experiment was repeated three times.

### Flow cytometry

The Annexin V-FITC Apoptosis Detection Kit (BD Bioscience, BectonDickinson Co., USA) was used to detect the apoptosis of cells. The HK-2 cells were treated with trypsin and collected by centrifugation, washed twice with pre-chilled PBS and then resuspended in 100 µL of 1× Binding Buffer at a concentration of about 1 × 10^6^ cells/mL. Then added 5 µL Annexin V-FITC and 10 µL PI staining solution, and mixed gently at room temperature for 10–15 min in the dark. 400 µL of 1×Binding Buffer was added to the above reaction sample, mixed and placed on ice. The sample was detected by flow cytometry by using a FACSCalibur flow cytometer (BD Biosciences, San Jose, CA, USA) within 1 h.

### Immunofluorescence assay

Cells were seeded on coverslips, fixed with paraformaldehyde (4 % in PBS 1×) and permeabilized with Triton X-100 solution (0.1 % in PBS 1×) for 10 min. To block non-specific binding, HK-2 cells were incubated with 10 % FBS in PBS 1× for 20 min, followed by incubation with primary antibody anti-LC3B (1:500) for 1 h at room temperature. After that, HK-2 cells were incubated with Alexa Fluor 488 anti-rabbit (1:1000) (Molecular Probes, A-11,034) secondary antibody for LC3. Finally, coverslips were mounted on microscope slides, by using fluoromount-G (SouthernBiotech, 0100–01) medium. Images were taken by using an inverted fluorescence microscope (Olympus, IX-51).

### Enzyme-linked immunosorbent assay (ELISA)

IL-6 and TNF-α concentration in the cultured supernatant were quantified by using the IL-6 ELISA kit, TNF-α ELISA Kit (Boster, Wuhan, China) according to the manufacturer’s instructions.

### Dual‐luciferase reporter assays

TargetScan (http://www.targetscan.org/vert_72/) analysis predicted the binding of miR-205 to the 3’- untranslated region (UTR) of PTEN. The target sequences of PTEN wild type 3’UTR and mutant 3’UTR were cloned into a pGL3-promoter luciferase vector (Promega, Madison, WI) which contained the Renilla luciferase gene. HEK-293T cells were co-transfected with mimic-NC or miR-205 mimic using Lipofectamine 2000 (Invitrogen). Cells were collected after 48 h for assay using the Dual Luciferase reporter assay system (Promega, Madison, WI). Values were normalized relative to Renilla luciferase activity.

### Animals and renal I/R injury

In this study, male 48 Sprague-Dawley rats (4–5 weeks old), weighing 180–220 g, were purchased from Beijing Vital River Laboratory Animal Technology Co., Ltd (Beijing, China). All rats were kept in a standard temperature-controlled room, with free access to water and a standard laboratory diet in a 12-h light /dark cycle. This study was approved by the Second Affiliated Hospital of Nanchang University Medical Research Ethics Committee Ethics Committee. SP1 overexpression plasmid or an equal volume of 0.9 % NaCl was administered via tail vein injection. Rats were anesthetized with intraperitoneal sodium pentobarbital (50 to 70 mg/kg) and placed on a homeothermic table to maintain core body temperature at 37°C. Both renal pedicles were occluded via a midline incision for 30 or 45 min followed by reperfusion for 3, 6, 12, 24 or 48 h. Sham surgery consisted of an identical procedure with the exception of application of the microaneurysm clamps. Scr was determined by standard picric acid reaction in serum obtained from the tail vein or via cardiac puncture.

### TUNEL staining

The paraffin sections with a thickness of 5 µm were taken for TUNEL staining. The changes in the nuclei of renal tissues and the positive expression of apoptotic nuclei stained to brown were observed under TUNEL staining light microscope (Nikon USA, Melville, New York) according to the manufacturer’s instructions, and the apoptosis rate of renal tubules was calculated.

### Immunohistochemical staining

Tissue sections were dewaxed in xylene for 30 min, rehydrated through graded alcohol to PBS, then inmmersed in 3 % hydrogen peroxide blocks endogenous peroxidase activity at room temperature. Next, the sections were placed in 0.01 mol/L sodium citrate buffer (PH6.0) and heated the autoclave to 100°C to repair the antigen. Then, 20 µL SP1 antibody (1: 250) was added and incubated overnight at 4°C. A biotinylated anti-rabbit secondary antibody was then added and incubated for 1 h and horseradish peroxidase streptavidin for 30 min at room temperature before being visualized with a DAB kit (Vector Laboratories, SK-4100). The sections were placed in hematoxylin staining solution for about 1 min and washed with water and then immediately placed in 0.1 % hydrochloric acid alcohol for about 5 s, washed with water, dehydrated with gradient alcohol, transparent xylene, and sealed with neutral gum. The percentage of stained target cells was evaluated in 10 random microscopic fields per tissue section, and their averages were subsequently calculated.

### Statistical analysis

The experimental results were analyzed by GraphPad Prism 8 software. All measurement data are presented as the mean ± standard deviation (SD), and statistical evaluation was performed using two-tailed Student’s *t-*test between two groups and one-way ANOVA test for more than three groups. Each experiment was performed at least three times. *P* < 0.05 was considered statistically significant.

## Results

### SP1 and miR-205 were downregulated, PTEN was upregulated in of HK-2 cells exposed to anoxia-reoxygenation injury

**Fig. 1:**
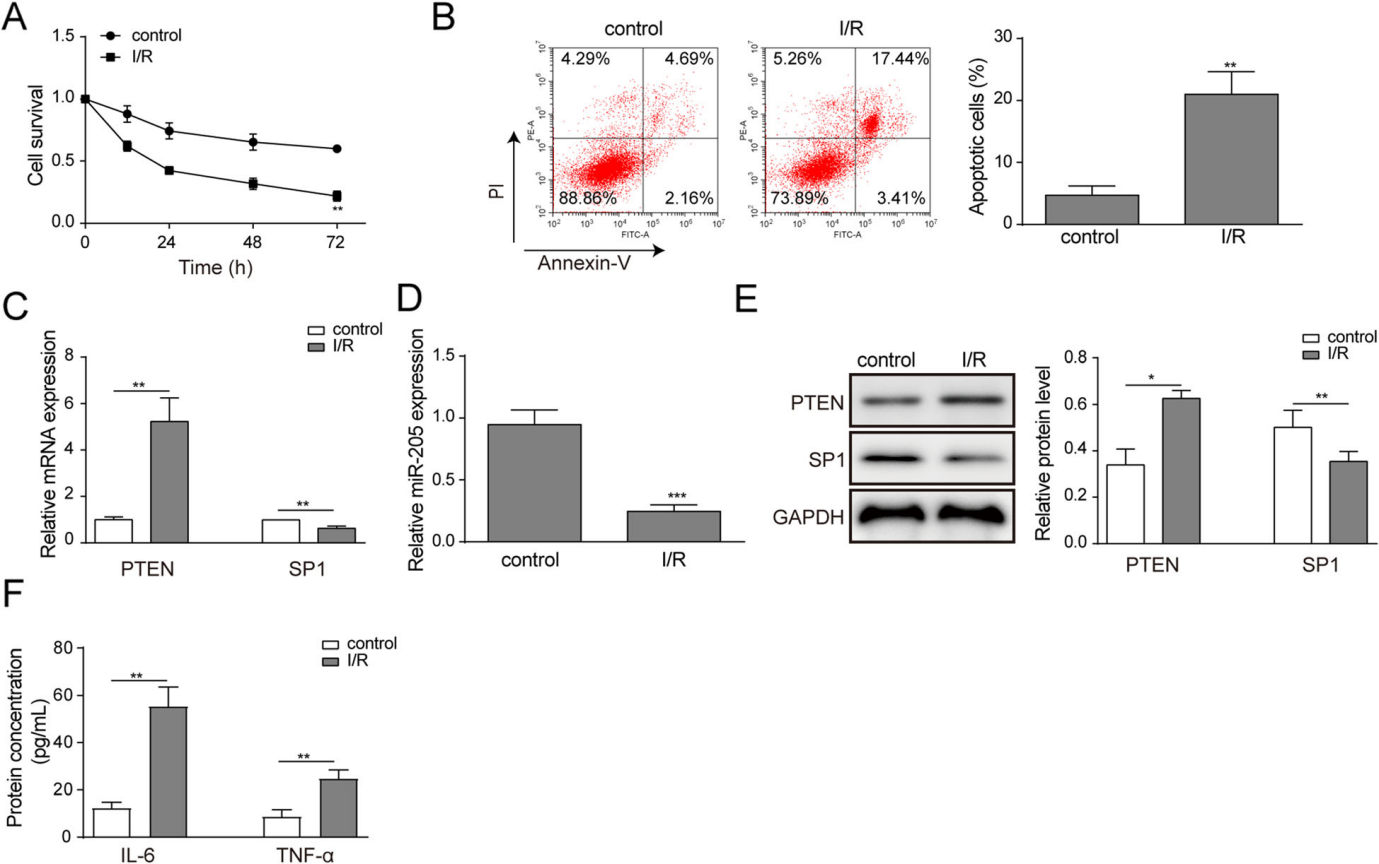
SP1 and miR-205 were downregulated, PTEN was upregulated in of HK-2 cells exposed to anoxia-reoxygenation injury. The HK-2 cells were treated with anoxia-reoxygenation injury. **a** Cell proliferation was determined by MTT. **b** Cell apotosis was detected by flow cytometry analysis. **c-d** The expression of SP1, miR-205 and PTEN detected were detected by RT-qPCR. **e** Western blot detection of SP1, PTEN protein expression. **f** The secretion of IL-6 and TNF-α were detected by ELISA. The data are expressed as the mean ± SD and are representative of 3 experiments. *P < 0.05, **P < 0.01, ***P < 0.001

We analyzed the proliferation changes of HK-2 cells with anoxia-reoxygenation injury by MTT assay. The results showed that the proliferation of HK-2 cells was significantly inhibited by anoxia-reoxygenation injury (Fig. 1a). The cell apoptosis was detected by flow cytometry and the apoptosis rate of HK-2 cells was significantly increased after anoxia-reoxygenation compared with those before anoxia (Fig. 1b). Then we performed the expression levels of SP1, PTEN and miR-205 by RT-qPCR in HK-2 cells with anoxia-reoxygenation injury. From the result, we found that the expressions of SP1 and miR-205 were decreased, while PTEN expression was significantly increased in HK-2 cells with anoxia-reoxygenation injury (Fig. 1c and d). Meanwhile, the protein expression levels of SP1 and PTEN were analyzed by western blot (Fig. 1e). The result showed that the protein expression levels of SP1 were decreased and that the protein expression levels of PTEN were increased in HK-2 cells with anoxia-reoxygenation injury. In addition, ELISA results showed that inflammatory factors IL-6 and TNF-α secretion were significantly increased in HK-2 cells with anoxia-reoxygenation injury, in comparison with the control (Fig. 1f).

### Overexpression of SP1 protected HK-2 cells against injury induced by anoxia‐reoxygenation

**Fig. 2:**
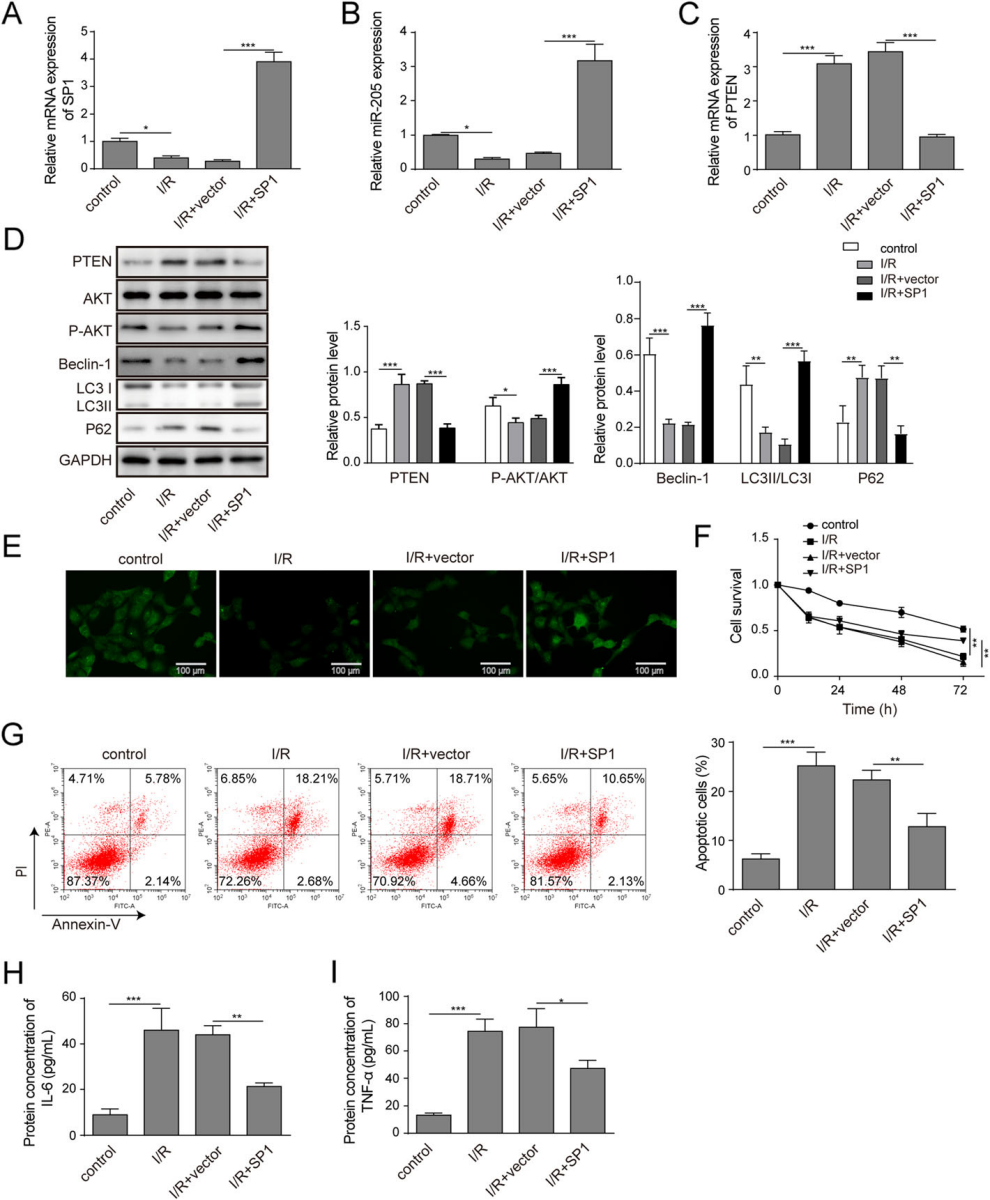
Overexpression of SP1 protected HK-2 cells against injury induced by anoxia-reoxygenation. The HK-2 cells were transfectd with SP1 overexpression vector. **a-c** RT-qPCR was used to detecte the expression of SP1, miR-205 and PTEN. **d** Western blot detected the protein expression of PTEN, AKT, p-AKT, Beclin-1,p62,LC3II and LC3I. **e** The autophagy protein LC3 expression localization was detected by immunofluorescence staining. **f** Cell proliferation was measured by MTT assay. **g** Flow cytometry analysis of cell apotosis. **h-i** The secretions of IL-6 and TNF-α were detected by ELISA. The data are expressed as the mean ± SD and are representative of 3 experiments. *P < 0.05, **P < 0.01, ***P < 0.001

To further investigate the role of SP1 in hypoxia-induced renal cell injury, the SP1 overexpression vector was transfected into HK-2 cells and the expression levels of SP1, miR-205 and PTEN were detected by RT-qPCR. After transfection of overexpression vector, the expression of SP1 mRNA in HK-2 cells were significantly increased and the overexpression of SP1 promoted miR-205 and inhibited PTEN expression (Fig. 2a and c). Western blot was then used to detect the expression of PTEN, AKT, p-AKT, autophagy-related proteins p62, LC3II and LC3I in HK-2 cells exposed to anoxia-reoxygenation injury. The results showed that the protein expression level of PTEN and p62 increased in HK-2 cells damaged by anoxia-reoxygenation injury, while the protein expression levels of p-AKT and LC3II/I were significantly decreased. Overexpression of SP1 could reduce the protein expression of PTEN and p62 and elevate the protein expression levels of p-AKT/AKT, Beclin-1 and LC3II/I (Fig. 2d). The results of immunofluorescence staining showed that the fluorescence intensity of autophagy protein LC3 was decreased in HK-2 cells after anoxia-reoxygenation, and the overexpression of SP1 could increase the autophagy protein LC3 fluorescence intensity (Fig. 2e). MTT results showed overexpression of SP1 could increase cell proliferation inhibited by anoxia-reoxygenation (Fig. 2f). Flow cytometry showed that overexpression of SP1 could reduce the apoptosis rate of HK-2 cells induced by I/R (Fig. 2g). Finally, ELISA results showed that overexpression of SP1 could reduce the secretion of inflammatory factors IL-6 and TNF-α induced by anoxia-reoxygenation (Fig. 2h and i).

### PTEN was a direct target of miR-205

**Fig. 3:**
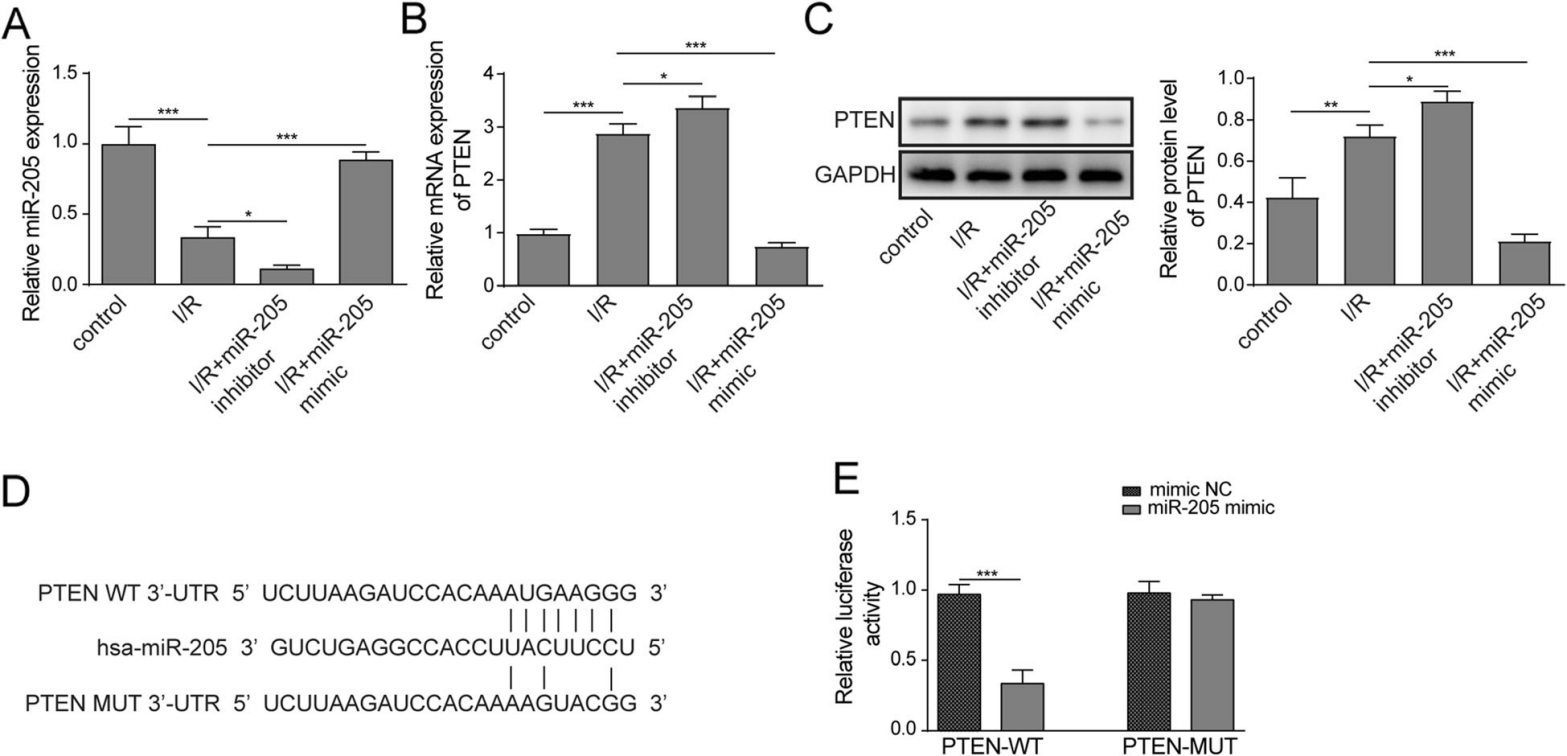
PTEN was a direct target of miR-205. The HK-2 cells were transfectd with miR-205 inhibitor or mimic. **a-b** The expression of miR-205 and PTEN was detected by RT-qPCR **c** The expression of PTEN was detected by RT-qPCR and western blot. **d-e** PTEN was a direct downstream target of miR-205 was confirmed by both bioinformatics target gene prediction and dual-luciferase report assay. The data are expressed as the mean ± SD and are representative of 3 experiments. *P < 0.05, **P < 0.01, ***P < 0.001

miR-205 inhibitor or mimic was transfected into HK-2 cells, and the transfection efficiency was confirmed by RT-qPCR. The level of miR-205 was significantly downregulated in cells transfected with the miR-205 inhibitor and miR-205 mimic upregulated the expression of miR-205 (Fig. 3a). The RT-qPCR and western blot detected PTEN expression level, which showed that miR-205 knockdown promoted PTEN expression, miR-205 overexpression inhibited PTEN expression (Fig. 3b and c). By using bioinformatics analytic tool (Targetscan), the 3’UTR of PTEN gene was found to be a target of miR-205 (Fig. 3d). To further verify the relationship between miR-205 and PTEN, the pGL3-luciferase reporter vectors of wild type (PTEN-WT) and mutant type (PTEN-MUT) 3′UTR of PTEN gene were successfully constructed. and dual-luciferase reporter assay showed that miR-205 mimic could significantly decrease the luciferase activity of PTEN-WT PTEN 3’-UTR plasmid, compared with the mutation plasmid transfection group (Fig. 3e).

### Overexpression of SP1 protected HK-2 cells injury induced by anoxia-reoxygenation via miR-205/PTEN/Akt pathway mediated autophagy

**Fig. 4:**
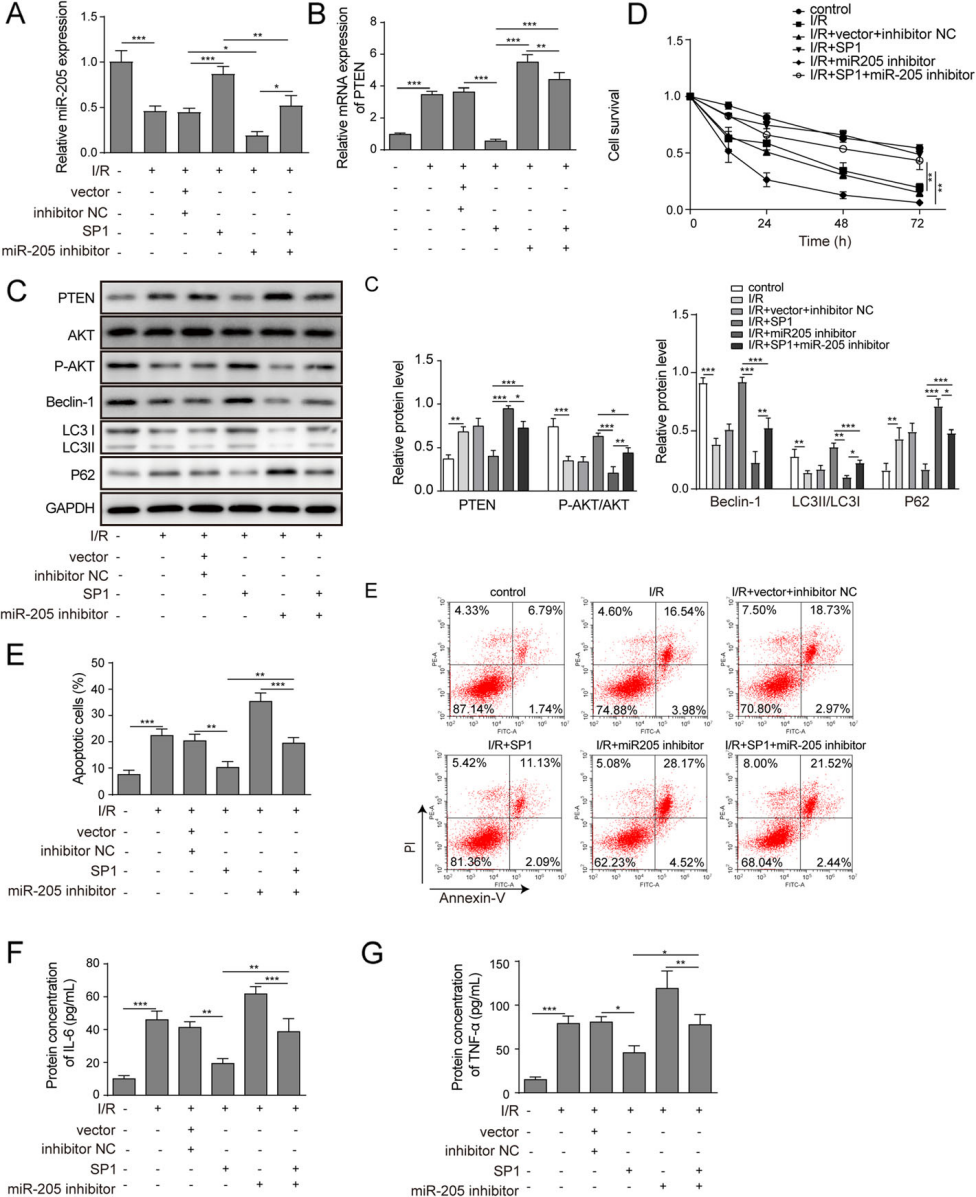
Overexpression of SP1 protected HK-2 cells injury induced by anoxia-reoxygenation via miR-205/PTEN/Akt pathway mediated autophagy. The HK-2 cells were transfectd with SP1 overexpression and miR-205 inhibitor. **a-b** The expression of SP1 and PTEN was detected RT-qPCR. **c** Western blot detection of PTEN, AKT, p-AKT, Beclin-1, p62,LC3II and LC31. **d** Cell proliferation measured by MTT assay. **e** Flow cytometry analysis of cell apotosis. **f-g** The secretion of IL-6 and TNF-α were detected by ELISA. The data are expressed as the mean ± SD and are representative of 3 experiments. *P < 0.05, **P < 0.01, ***P < 0.001

The SP1 overexpression vector and miR-205 inhibitor were transfected into HK-2 cells, and the expression levels of miR-205 and PTEN were determined by RT-qPCR. The results showed that overexpression of SP1 could promote miR-205 expression and inhibit PTEN expression in cells induced by anoxia-reoxygenation, while miR-205 inhibitor could reverse the expression of miR-205 promoted by overexpression of SP1 and inhibit PTEN expression in HK-2 cells induced by anoxia-reoxygenation (Fig. 4a and b). Western blot was then used to detect protein expression levels of PTEN, AKT, p-AKT, Beclin-1, p62, LC3II and LC3I. The results showed that overexpression of SP1 inhibited the expression of PTEN and p62 protein and promoted the protein expression of p-AKT/AKT, Beclin-1 and LC3II/I. The miR-205 inhibitor reversed the effect of SP1 overexpression, which promoted PTEN and p62 protein expression, and inhibited p-AKT/AKT, Beclin-1 and LC3II/I protein levels (Fig. 4c). MTT assay showed that overexpression of SP1 promoted cell proliferation inhibited by anoxia-reoxygenation, and that miR-205 inhibitor reversed the effect of SP1 overexpression to inhibit cell proliferation of HK-2 cells (Fig. 4d). Flow cytometry was used to detect the rate of apoptosis and the result found that overexpression of SP1 inhibited apoptosis, and miR-205 inhibitor reversed the effect of SP1 overexpression to promote apoptosis of HK-2 cells induced by anoxia-reoxygenation (Fig. 4e). ELISA assay showed that SP1 overexpression could inhibit the secretion of IL-6 and TNF-α, miR-205 inhibitor could reverse the effect of SP1 overexpression to promote the secretion of IL-6 and TNF-α from HK-2 cells induced by anoxia-reoxygenation (Fig. 4f and g).

### Overexpression of SP1 attenuated renal I/R injury in rats

**Fig. 5:**
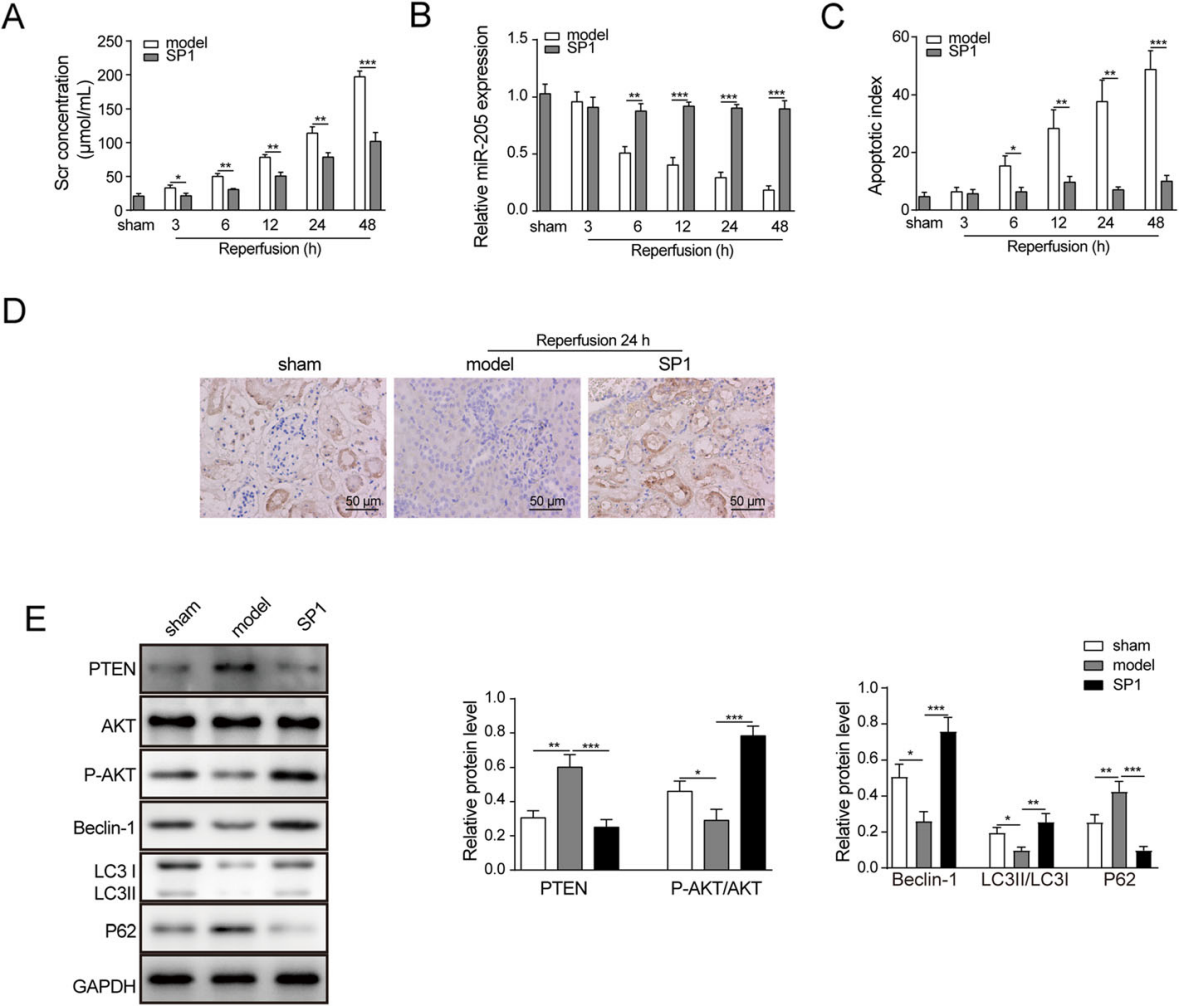
Overexpression of SP1 attenuated renal ischemia-reperfusion injury in rats. A rat model of renal ischemia-reperfusion injury was prepared and treated with a SP1 overexpression vector. **a** Detection of Scr levels by ELISA. **b** RT-qPCR was used to detect the expression of miR-205. **c** TUNEL staining was used to detect cell apoptosis. **d** Immunohistochemical detection of SP1 expression distribution. **e** Western blot detected the expression of PTEN, AKT, p-AKT, Beclin-1, p62, LC3II and LC31. The data are expressed as the mean ± SD and are representative of 3 experiments. *P < 0.05, **P < 0.01, ***P < 0.001

To observe the effect of SP1 on renal I/R injury and its mechanism *in vivo*, a rat model of renal I/R injury was prepared and SP1 overexpression vector was used to treat these rats. The results of the detection of serum creatinine (Scr) levels, an indicator of liver function, showed that the levels of Scr in serum was increased significantly after I/R in rats, reaching the peak at 48 h. Overexpression of SP1 could significantly reduce the secretion of Scr (Fig. 5a). Then, RT-qPCR was used to detect the expression of miR-205 in renal tissues. The results showed that the expression of miR-205 in kidney tissues of rats was significantly reduced after I/R, and overexpression of SP1 could significantly promote the expression of miR-205 (Fig. 5b). TUNEL staining was used to detect the apoptosis and the results showed that apoptotic index significantly increased after I/R in the renal tissues of rats, and overexpression of SP1 could inhibit the apoptosis (Fig. 5c). The results of immunohistochemical analysis showed that SP1 expression in kidney tissues was decreased after I/R, and the expression of SP1 was increased after overexpression of SP1 (Fig. 5d). Western blot analysis showed that the expression of PTEN and p62 were increased after I/R in rat kidney tissue, while the protein expression levels of p-AKT/AKT, Beclin-1 and LC3II/LC3I were decreased significantly. Overexpression of SP1 could inhibit the expression of PTEN and p62 protein in rat kidney tissue, and promote the expression of p-AKT/AKT, Beclin-1 and LC3II/LC3I (Fig. 5e).

## Discussion

Renal tubular epithelium is the most sensitive cell to renal ischemia, and the loss and damage of renal tubular epithelial cells is an important cause of renal dysfunction during injury [22]. Recently, research on renal ischemic injury has focused on the role of various specific molecular substances in renal tubular ischemia or early reperfusion in the repair of renal injury [23]. The mechanism of renal I/R injury is generally believed to have the following mechanisms, that is, glandular acid metabolism disorder, oxygen free radical effect, acidosis, calcium metabolism disorder, lipid peroxidation damage, mitochondrial damage and microvascular damage [24, 25]. Our research took renal tubular epithelial cells as the research object, performed in vitro simulation of ischemic injury, and explored its possible molecular mechanism.

Autophagy is a conservative, multi-step approach that maintains cell homeostasis by degrading and circulating damaged organelles and macromolecules. Autophagy pathways were briefly upregulated under stress conditions such as cell starvation, hypoxia, deprivation of nutrients and growth factors, endoplasmic reticulum stress, oxidative damage, and most of them are involved in the pathogenesis of AKI [26, 27]. Pharmacological and genetic inhibition studies have shown that autophagy played a renal protective role in AKI [28]. However, the role of autophagy in renal recovery and repair after AKI remains unclear. In many studies, the dynamic changes of autophagy were of great significance to the proliferation and repair of tubules during the recovery period of AKI [29, 30]. Increasing evidence suggests that autophagy was closely related to kidney health and disease [31, 32]. Our results further confirmed that ischemic injury led to a reduction in HK-2 cell autophagy. Up-regulation of autophagy by changing the expression of related genes improves cell damage.

More and more evidences show that miRNAs regulate autophagy in various cell types by targeting autophagy-related genes. MiR-221/222 from exosomes of human aortic smooth muscle cells (HAoSMCs) inhibits autophagy in human umbilical vein endothelial cells (HUVEC) by modulating the PTEN/Akt signaling pathway [33]. Under ultraviolet irradiation, overexpression of miR-205-3p could increase the viability and proliferation ability of human corneal epithelial (HCE) cells, and reduce the apoptosis and autophagy ability of HCE cells [34]. The growth inhibition triggered by mir-205 in nasopharyngeal carcinoma was mainly due to the induction of autophagy, and was related to the increase of LC3B II and the decrease of p62 expression [35]. Study showed that miR-205 directly targeted PTEN, regulated Akt/autophagy pathway and MMP2 expression, and played a key role in the function of endothelial progenitor cells (EPCs), deep vein thrombosis (DVT) recanalization and regression [36]. The PTEN/Akt signaling pathway mediated the transition from LC3I to LC3II during autophagy to regulate breast cancer cell proliferation [37]. Our results revealed that SP1 regulated miR-205/PTEN axis and mediated Akt signaling pathway to regulate autophagy and improve acute renal cell injury induced by ischemia-reperfusion. We also verified that overexpression of SP1 could reduce the secretion of Scr, inhibit cell apoptosis and promote autophagy to attenuate renal ischemia-reperfusion injury in rats.

AKI is one of the most common and serious clinical diseases. Although we have a full understanding of the pathophysiological mechanism of AKI, the treatment and prognosis of AKI have not made significant progress in recent years [29]. Studies have shown that in AKI, renal tubular cells can be protected by inducing autophagy [38]. After acute kidney injury, the autophagy response is increased and it protects the kidneys. In the recovery period of acute kidney injury, autophagy could promote cell proliferation and thus to promote the regeneration and repair of renal tubules. The dual role of autophagy was played in acute kidney injury and repair. On the one hand, the continuous activation of autophagy may cause renal tubular atrophy, thereby promoting renal fibrosis. On the other hand, autophagy could protect against fibrosis by degrading excessive collagen in cells [39]. The role will be helpful for the treatment of acute kidney injury and prevention of its progress. Our study clarified that SP1 promotes autophagy through miR-205/PTEN/Akt, and provided a potential target for the treatment of acute renal cell injury caused by I/R. However, the specific regulation mechanism is still not clear. In future research, we will further explore the specific regulation mode between molecules to provide a clearer theoretical basis for the clinical treatment of AKI.

## Conclusions

In summary, we found that overexpression of SP1 overexpression could restore autophagy through miR-205/PTEN/Akt pathway and repair the damage- re-implantation- induced acute kidney injury. Our study provided the molecular mechanism of SP1-regulated autophagy in the treatment of AKI. Targeting the SP1/miR-205/PTEN/Akt axis could as a potential therapeutic strategy for AKI induced by ischemia-reperfusion.

## Data Availability

All data generated or analyzed during this study are included in this article. The datasets used and/or analyzed during the current study are available from the corresponding author on reasonable request.

## References

[1] Gameiro J, Branco T, Lopes JA. Artificial Intelligence in Acute Kidney Injury Risk Prediction. J Clin Med. 2020;9(3). 10.3390/jcm9030678 PMC7141311 32138284

[2] Rosner MH, La Manna G, Ronco C (2018). Acute Kidney Injury in the Geriatric Population. Contrib Nephrol.

[3] Odutayo A, Wong CX, Farkouh M, Altman DG, Hopewell S, Emdin CA (2017). AKI and Long-Term Risk for Cardiovascular Events and Mortality. J Am Soc Nephrol.

[4] Vanmassenhove J, Kielstein J, Jorres A, Biesen WV (2017). Management of patients at risk of acute kidney injury. Lancet.

[5] Matsumoto T, Urushido M, Ide H, Ishihara M, Hamada-Ode K, Shimamura Y (2015). Small Heat Shock Protein Beta-1 (HSPB1) Is Upregulated and Regulates Autophagy and Apoptosis of Renal Tubular Cells in Acute Kidney Injury. PLoS One.

[6] Hesketh EE, Czopek A, Clay M, Borthwick G, Ferenbach D, Kluth D, et al. Renal ischaemia reperfusion injury: a mouse model of injury and regeneration. J Vis Exp. 2014(88). 10.3791/51816 PMC4188040 24961244

[7] Chen Y, Jiang S, Zou J, Zhong Y, Ding X (2016). Silencing HIF-1alpha aggravates growth inhibition and necrosis of proximal renal tubular epithelial cell under hypoxia. Ren Fail.

[8] Bansal S, Patel RN (2020). Pathophysiology of Contrast-Induced Acute Kidney Injury. Interventional cardiology clinics.

[9] Scurt FG, Bose K, Canbay A, Mertens PR, Chatzikyrkou C (2020). [Acute kidney injury following acute pancreatitis (AP-AKI): Definition, Pathophysiology, Diagnosis and Therapy]. Z Gastroenterol.

[10] Matsushita K, Saritas T, Eiwaz MB, McClellan N, Coe I, Zhu W (2020). The acute kidney injury to chronic kidney disease transition in a mouse model of acute cardiorenal syndrome emphasizes the role of inflammation. Kidney Int.

[11] Xie Y, Xiao J, Fu C, Zhang Z, Ye Z, Zhang X (2018). Ischemic Preconditioning Promotes Autophagy and Alleviates Renal Ischemia/Reperfusion Injury. Biomed Res Int.

[12] Decuypere JP, Ceulemans LJ, Agostinis P, Monbaliu D, Naesens M, Pirenne J (2015). Autophagy and the Kidney: Implications for Ischemia-Reperfusion Injury and Therapy. Am J Kidney Dis.

[13] Price PM, Safirstein RL, Megyesi J (2009). The cell cycle and acute kidney injury. Kidney Int.

[14] Vizcaíno C, Mansilla S, Portugal J (2015). Sp1 transcription factor: A long-standing target in cancer chemotherapy. Pharmacol Ther.

[15] O’Connor L, Gilmour J, Bonifer C (2016). The Role of the Ubiquitously Expressed Transcription Factor Sp1 in Tissue-specific Transcriptional Regulation and in Disease. Yale J Biol Med.

[16] Beyazit H, Demiryürek AT, Temel MT, Pekpak E, Demiryürek S, Akbayram S. Investigation of Dynamic Thiol/Disulfide Homeostasis in Children With Acute Immune Thrombocytopenia. J Pediatr Hematol Oncol. 2019. 10.1097/MPH.0000000000001494 31033791

[17] Yuan X, Li D, Chen X, Han C, Xu L, Huang T (2017). Extracellular vesicles from human-induced pluripotent stem cell-derived mesenchymal stromal cells (hiPSC-MSCs) protect against renal ischemia/reperfusion injury via delivering specificity protein (SP1) and transcriptional activating of sphingosine kinase 1 and inhibiting necroptosis. Cell Death Dis.

[18] Wang H, Li K, Mei Y, Huang X, Li Z, Yang Q (2018). Sp1 Suppresses miR-3178 to Promote the Metastasis Invasion Cascade via Upregulation of TRIOBP. Mol Ther Nucleic Acids.

[19] Pan F, Mao H, Bu F, Tong X, Li J, Zhang S, et al. Sp1-mediated transcriptional activation of miR-205 promotes radioresistance in esophageal squamous cell carcinoma. Oncotarget. 2016. 10.18632/oncotarget.13902 PMC5351585 27974696

[20] Zhang P, Lu X, Shi Z, Li X, Zhang Y, Zhao S (2019). miR-205-5p regulates epithelial-mesenchymal transition by targeting PTEN via PI3K/AKT signaling pathway in cisplatin-resistant nasopharyngeal carcinoma cells. Gene.

[21] Chen W, Ruan Y, Zhao S, Ning J, Rao T, Yu W (2019). MicroRNA-205 inhibits the apoptosis of renal tubular epithelial cells via the PTEN/Akt pathway in renal ischemia-reperfusion injury. Am J Transl Res.

[22] Linkermann A, Brasen JH, Himmerkus N, Liu S, Huber TB, Kunzendorf U (2012). Rip1 (receptor-interacting protein kinase 1) mediates necroptosis and contributes to renal ischemia/reperfusion injury. Kidney Int.

[23] Zhou Y, Cai T, Xu J, Jiang L, Wu J, Sun Q (2017). UCP2 attenuates apoptosis of tubular epithelial cells in renal ischemia-reperfusion injury. Am J Physiol Renal Physiol.

[24] Liu XJ, Tan Y, Geng YQ, Wang Z, Ye JH, Yin XY (2014). Proximal tubule toll-like receptor 4 expression linked to inflammation and apoptosis following hypoxia/reoxygenation injury. Am J Nephrol.

[25] Arai S, Kitada K, Yamazaki T, Takai R, Zhang X, Tsugawa Y (2016). Apoptosis inhibitor of macrophage protein enhances intraluminal debris clearance and ameliorates acute kidney injury in mice. Nat Med.

[26] Mei S, Livingston M, Hao J, Li L, Mei C, Dong Z (2016). Autophagy is activated to protect against endotoxic acute kidney injury. Sci Rep.

[27] Kaushal GP, Shah SV (2016). Autophagy in acute kidney injury. Kidney Int.

[28] Zhu L, Yuan Y, Yuan L, Li L, Liu F, Liu J (2020). Activation of TFEB-mediated autophagy by trehalose attenuates mitochondrial dysfunction in cisplatin-induced acute kidney injury. Theranostics.

[29] Cui J, Bai X, Chen X (2020). Autophagy and Acute Kidney Injury. Adv Exp Med Biol.

[30] Gong L, Pan Q, Yang N (2020). Autophagy and Inflammation Regulation in Acute Kidney Injury. Front Physiol.

[31] Tracz MJ, Juncos JP, Croatt AJ, Ackerman AW, Grande JP, Knutson KL (2007). Deficiency of heme oxygenase-1 impairs renal hemodynamics and exaggerates systemic inflammatory responses to renal ischemia. Kidney Int.

[32] Jiang M, Liu K, Luo J, Dong Z (2010). Autophagy is a renoprotective mechanism during in vitro hypoxia and in vivo ischemia-reperfusion injury. Am J Pathol.

[33] Li L, Wang Z, Hu X, Wan T, Wu H, Jiang W (2016). Human aortic smooth muscle cell-derived exosomal miR-221/222 inhibits autophagy via a PTEN/Akt signaling pathway in human umbilical vein endothelial cells. Biochem Biophys Res Commun.

[34] Fu JY, Yu XF, Wang HQ, Lan JW, Shao WQ, Huo YN (2020). MiR-205-3p protects human corneal epithelial cells from ultraviolet damage by inhibiting autophagy via targeting TLR4/NF-kappaB signaling. Eur Rev Med Pharmacol Sci.

[35] Hao Y, Li J, Zhang H, Guan G, Guo Y (2020). MicroRNA-205 targets HER3 and suppresses the growth, chemosensitivity and metastasis of human nasopharyngeal carcinoma cells. J BUON.

[36] Sun LL, Xiao L, Du XL, Hong L, Li CL, Jiao J (2019). MiR-205 promotes endothelial progenitor cell angiogenesis and deep vein thrombosis recanalization and resolution by targeting PTEN to regulate Akt/autophagy pathway and MMP2 expression. J Cell Mol Med.

[37] Wang X, Li Y, Fan Y, Yu X, Mao X, Jin F (2018). PTBP1 promotes the growth of breast cancer cells through the PTEN/Akt pathway and autophagy. J Cell Physiol.

[38] Liu Y, Xiao J, Sun J, Chen W, Wang S, Fu R (2020). ATG7 promotes autophagy in sepsisinduced acute kidney injury and is inhibited by miR526b. Mol Med Rep.

[39] Duann P, Lianos EA, Ma J, Lin PH. Autophagy. Innate Immunity and Tissue Repair in Acute Kidney Injury. Int J Mol Sci. 2016;17(5). 10.3390/ijms17050662 PMC4881488 27153058

